# 

**Published:** 1859-11

**Authors:** 


NORTH AMERICAN

### MEDICO-CHIRURGICAL REVIEW.

Vol. III.] NOVEMBER, 1859. [No. 6.

### CONTENTS.

### ANALYTICAL AND CRITICAL REVIEWS.

ART. PAGE

I.—Manual Complet de Médecine Légales. By J. BRIAND, M.D., and ERNEST CHAUDÉ, Counselor-at-law, etc. . . . . 961

II.—The Diseases of the Stomach; with an Introduction on its Anatomy and Physiology. By WILLIAM BRINTON, M.D. . . . 1000

III.—1. A Treatise on Gonorrhœa and Syphilis. By SILAS DURKEE, M.D. . . . . . . . . . . . . . 1015

2. A Treatise on the Venereal Disease. By PHILIP RICORD, M.D. 1015

IV.—Anatomy, Descriptive and Surgical. By HENRY GRAY, F.R.S. . 1022

V.—Leçons Theoriques et Cliniques sur les Affections Cutanées Parasitaires. By Dr. BAZIN. . . . . . . . . . 1026

VI.—The Use and Abuse of Tobacco. By JOHN LIZARS. . . . . 1029

VII.—A Guide to the Practical Study of the Diseases of the Eye. By JAMES DIXON, F.R.C.S. . . . . . . . . . 1032

VIII.—A Practical Treatise on the Diagnosis, Pathology, and Treatment of the Diseases of the Heart. By AUSTIN FLINT, M.D. . 1033

### ORIGINAL COMMUNICATIONS.

I.—Contributions to Obstetric Jurisprudence. (No. 6.) By HORATIO R. STORER, M.D. . . . . . . . . . . . 1033

II.—Some Remarks on Cardiac Hypertrophy and Dilatation. By AUSTIN FLINT, M.D. . . . . . . . . . . . 1046

III.—A Case of Laceration of the Uterus. By L. BEECHER TODD, M.D. 1052

IV.—A Case of Stomatitis Materna Cured by the Syrup of the Phosphates. By J. C. REEVE, M.D. . . . . . . . . 1053

V.—Proceedings of the Pathological Society of Philadelphia. . . 1056

### BI-MONTHLY ABSTRACTS.

#### SURGERY.

I.—Aneurism of the Ischiatic Artery. . . . . . . . . 1066

II.—On Section of the Supra-Orbital Nerve in Spasms of the Eyelids. 1067

III.—New Treatment of Hydrocele. . . . . . . . . 1067

IV.—On Bloody Tumors of the Pavilion of the Ear, in the Insane. . 1068

V.—Report on Amputation of the Penis for Epithelial Cancer. . 1068

VI.—On Buccal Cancer in Smokers. . . . . . . . . . 1069

VII.—On the Treatment of Spina Bifida by Injections of Iodine. . 1070

VIII.—On Local Sweating of the Eyelids. . . . . . . . 1071

#### PHYSIOLOGY.

I.—On the Natural Constants of the Healthy Urine of Man. . . 1071

II.—On some Actions performed by Voluntary Muscles. . . . 1072

III.—Observations on the Action of Pancreatic Juice on Albumen. . 1073

IV.—On the Effects of Direct Excitation of the Liver and Kidney. . 1073

V.—On the Reactions of the Medullary Substance of the Supra-Renal Capsules. . . . . . . . . . . . . 1074

**

ART. PAGE
VI.—Notes on the Phenomena which occur in the Tails of the very young Embryo of Frogs, when they have been detached from the Body. . . . . . . . . . . 1074

PATHOLOGY AND THERAPEUTICS.

I.—On Fatal Steatosis of the Liver and Kidney. . . . . . 1076
II.—On Abscess in the Anterior Mediastinum. . . . . . . 1078
III.—On the Treatment of Chorea by Cauterization. . . . . . 1079
IV.—Professor Skoda's Formula in Scorbutus. . . . . . . 1080
V.—Saline Injections in Diphtheritis. . . . . . . . . 1080

OBSTETRICS, AND DISEASES OF WOMEN AND CHILDREN.

I.—Amputation of the Shoulder-Blade as a Method of Embryotomy. 1080
II.—Contributions to the History of Extra-Uterine Pregnancies. . 1081
III.—On the Induction of Premature Labor by Uterine Catheterism. . 1084
IV.—On the Treatment of Uterine Hemorrhage by Ruta and Sabina. 1085
V.—A Convenient Mode of Treating Vaginitis and Superficial Inflammation of the Cervix Uteri. . . . . . . . 1085
VI.—On the Influence of Sex on Diseases of Children. . . . 1086
VII.—Prophylactic Treatment of the Sequelæ of Measles and Scarlet Fever. . . . . . . . . . . 1086
VIII.—On the Treatment of Hooping-Cough. . . . . . . 1087
IX.—On the Curability of Tubercular Meningitis. . . . . 1087
X.—Defective Assimilation in Infants. . . . . . . . 1089

### BI-MONTHLY PERISCOPE.

SURGERY.

1. Excision of the Testis for Malignant Disease. . . . . . . 1090
2. On the Diagnosis of the Sacro-Iliac Disease. . . . . . 1093
3. Report of a Case of Extirpation of the Tongue. . . . . . 1095
4. Division of the Popliteal Nerve for Neuralgia of the Leg. . . 1099
5. Case of Spina Bifida treated by Collodion. . . . . . . 1100
6. On Foreign Bodies in the Urethra and Bladder. . . . . . 1101

PRACTICE OF MEDICINE.

1. Cases of Chorea treated by Sulphate of Zinc. . . . . . 1104
2. On the Hypodermic Treatment of Diseases. . . . . . 1110
3. On Some Points in the Treatment and Clinical History of Asthma. . 1117
4. A Case of Hæmoptysis. . . . . . . . . . . . 1123
5. On Puncture of Hydatid Cysts of the Liver with the Capillary Trocar. 1124

EDITORS' TABLE.—Fees for Professional Services. . . . . . . 1125
Letters of Advice. . . . . . . . . . . . . 1129
Oration on Harvey.—University of the Pacific.—New Medical Journal. . . . . . . . . . . . . . . 1130
The Pathological Society of Philadelphia.—The New Sydenham Society.—Reminiscences of Samuel Latham Mitchill, M.D., LL.D. 1131
Clinical Reports.—Close of the Third Volume of the Review. . 1133

NECROLOGICAL NOTICES.—Dr. William Pulteney Alison. . . . . 1133
Mr. Brunel. . . . . . . . . . . . . . . 1134

BI-MONTHLY BIBLIOGRAPHICAL RECORD. . . . . . . . . 1136

Index to Volume III. . . . . . . . . . . . . . 1137

